# Frequency and correlates of driving status among the oldest old: results from a large, representative sample

**DOI:** 10.1007/s40520-022-02252-3

**Published:** 2022-09-19

**Authors:** André Hajek, Hans-Helmut König

**Affiliations:** Department of Health Economics and Health Services Research, Hamburg Center for Health Economics, University Medical Center Hamburg-Eppendorf, Hamburg, Germany

**Keywords:** Oldest old aged, 80 and over, Driving habits, Automobile driving, Car, Frequency, Driving status

## Abstract

**Background/aims:**

In the light of the restricted knowledge, our aim was to explore the frequency and correlates of driving status among the oldest old.

**Methods:**

Data came from the representative "Survey on quality of life and subjective well-being of the very old in North Rhine-Westphalia (NRW80 +)” consisting of community-dwelling and institutionalized individuals ≥ 80 years residing in the most populous German state (North Rhine-Westphalia; *n* = 1,832 in the analytical sample, mean age: 86.5 years). The present driving status (no; yes, I drive myself; yes, as a passenger; yes, as driver and passenger) served as outcome measure.

**Results:**

Overall, 43.62% (95% CI 40.98–46.29%) of the individuals did not drive, whereas 30.12% (95% CI 27.75–32.59%) of the individuals drove by themselves, 20.97% (95% CI 18.91–23.20%) of the individuals drove as a passenger, and 5.29% of the individuals drove both (by themselves and as a passenger) (95% CI 4.16–6.71%). Multinomial logistic regressions showed, e.g., that being male (RRR: 0.13, 95% CI 0.09–0.18), younger age (RRR: 0.88, 95% CI 0.84–0.91), being married and living together with spouse (RRR: 1.48, 95% CI 1.08–2.02), living in a private household (RRR: 0.04, 95% CI 0.01–0.35), better self-rated health (RRR: 1.26, 95% CI 1.02–1.56), and lower functional impairment (RRR: 19.82, 95% CI 12.83–30.62) were positively associated with ‘Yes, I drive myself’ (compared to not driving a car).

**Discussion/conclusions:**

A sizable proportion of the individuals aged 80 years and above still drove by themselves. Less than half of the oldest old individuals did not drive. Moreover, our current study identified some correlates of driving status among individuals in latest life.

## Introduction

Changes in demographic composition, i.e., an increase in the number of individuals aged 80 years and over, are expected in the coming decades. In accordance with this growth, the number of older people with a driver's license will presumably rise [1]. A recent systematic review and meta-analysis showed that older drivers are at increased risk of fatal crash involvement [2]—which particularly relates to fragility [3] and sometimes inappropriate safety measures for older individuals [4]. On the other side, it can assist in, among other things, maintaining autonomy in late life. For all of these reasons, it is important to identify the frequency and the correlates of driving status among the oldest old.

While some studies (e.g., [5–7]) exist investigating the frequency and correlates of driving behavior in old age, there are only very few studies examining the frequency and correlates of driving behavior among the oldest old (i.e., aged 80 years and over) [8, 9]. For example, one study used data from the “1921–26 cohort of the Australian Longitudinal Study on Women’s Health” (wave 6 with *n* = 4025 women, mean age of 86.7 years, 85–90 years) [9]. The proportion of women who were still driving equaled 36.2% in this former study [9]. This recent study also identified several correlates of driving in regression analysis (namely: caring for others, living in rural areas of Australia, volunteering activities, living alone, having a higher educational level, and social interactions) [9]. Due to the very limited knowledge, our aim of this current study was to investigate the frequency and correlates of driving status among the oldest old.

Such knowledge is important, because driving status can contribute to health-related quality of life among the oldest old [10]. In addition, driving in advanced age can maintain mobility, autonomy, and social engagement. Driving a car may also be related to things like having access to goods and services, engaging in activities outside of the home, and preserving social connections, especially among individuals in this age bracket [11, 12]. To put it the other way around: transport poverty (i.e., “the social exclusion of marginalized individuals […] who do not have access to public or private transportation”, [13] p. 2) can have harmful consequences for the subjective well-being of individuals [14].

## Methods

### Sample

Data for this study were gathered from the “Survey on quality of life and subjective well-being of the very old in North Rhine-Westphalia (NRW80 +)”. This study was conducted in North Rhine-Westphalia from August 2017 to February 2018 which is the most populous state in Germany. The NRW80 + study is representative of individuals living in North Rhine-Westphalia aged ≥ 80 years (men and individuals ≥ 85 years were oversampled; therefore, weights were applied). Various topics were covered such as socio-economic or health-related issues. The key inclusion criteria were: a registered principal residence in North Rhine-Westphalia. This includes individuals in institutionalized surroundings and individuals living in private homes. The response rate was 23.4%. Nevertheless, main sociodemographic factors, such as age bracket, sex, or living conditions, were not associated with the probability of non-response [15]. In total, 1,863 individuals took part. Our analytical sample (for regression analysis) equaled 1,832 individuals due to a few missing values in the independent variables.

### Outcome measures: driving status

The present driving status served as outcome measure. It was distinguished between: no; yes, I drive myself; yes, as a passenger; yes, as driver and passenger. This is a common way to assess the driving status in large studies.

In a sensitivity analysis, we trichotomized the outcome (0 = no; 1 = yes, I drive myself or yes, as driver and passenger; 2 = yes, as a passenger).

### Independent variables

In multinomial logistic regression analysis, sociodemographic variables were used as follows: sex, age, and family situation (married; other (married, living separated from spouse; divorced; single; widowed). Additionally, in regression analysis, these health-related factors were included: self-rated health (single item, from 1 = very bad to 4 = very good), functional impairment, and the number of chronic conditions. In sum, 19 chronic conditions were included (no = 0, yes = 1; in each case): myocardial infarction, heart failure, hypertension, stroke, mental illness, cancer, diabetes, respiratory or pulmonary disease, back pain, gastric or intestinal disease, kidney disease, liver disease, blood disease, joint or bone disease, bladder disease, sleep disorder, eye disease or visual disorder, ear disease or hearing impairment, and neurological disease. A count score was generated (higher number reflects thus a higher number of chronic conditions). Moreover, a modified Lawton and Brody IADL tool [16] was used to measure functional impairment (in each case: 0 = only possible with help to 2 = no help required). By averaging the seven items, a score was generated (ranging from 0 to 2, with higher values corresponding to lower functional impairment).

### Statistical analysis

In a first step, sample characteristics for the analytical sample are depicted. Additionally, the frequency of driving status is displayed stratified by age group and sex. Thereafter, multiple multinomial logistic regressions are estimated to explore the correlates of driving status (with “no” as base outcome). The level of significance was set at α = 0.05. Statistical analysis was performed using Stata Release 16.1 (Stata Corp., College Station, Texas).

## Results

### Sample characteristics

Descriptive findings for our analytical sample are shown in Table 1. Average age was 86.5 years (SD: 4.5 years, 80–102 years). About 50.1% of the individuals were female. Additional details are provided in Table 1.

**Table 1:** Sample characteristics (for the analytical sample) (*n* = 1832)

	Mean (SD)/*N *(%)
Sex	
Men	915 (49.9%)
Women	917 (50.1%)
Age	86.5 (4.5)
Marital status	
Married, living separated from spouse; widowed; divorced; single	1088 (59.4%)
Married	744 (40.6%)
Living situation	
Living in a private household	1690 (92.2%)
Living in an institutionalized setting	142 (7.8%)
Self-rated health (ranging from 1 = very bad to 4 = very good)	2.6 (0.8)
Functional impairment (IADL; ranging from 0 to 2, with higher values corresponding to lower functional impairment)	1.4 (0.7)
Count score: number of chronic conditions (from 0 to 19)	3.5 (2.3)

In sum, 43.62% (95% CI 40.98–46.29%) of the individuals did not drive, whereas 30.12% (95% CI 27.75–32.59%) of the individuals drove by themselves, 20.97% (95% CI 18.91–23.20%) of the individuals drove as a passenger, and 5.29% of the individuals drove both (by themselves and as a passenger) (95% CI 4.16–6.71%).

In Table 2, the driving status (by sex and age group) is displayed. The frequency of driving largely varied by age group and sex. For example, the proportion of male individuals aged 80 to 84 years driving by themselves equaled 59.1%, whereas the proportion was 28.7% among female individuals in the same age bracket. The proportion markedly dropped to about 23.0% among male individuals aged 90 years and older (female individuals in this age bracket: 2.0%).

**Table 2:** Frequency: driving status (by sex and age group)

Driving status	Men			Women		
	80–84 years	85–89 years	90 years and older	80–84 years	85–89 years	90 years and older
	*N* = 384	*N* = 299	*N* = 244	*N* = 344	*N* = 326	*N* = 266
No: % (95% CI)	19.59% (15.61–24.29%)	30.49% (24.96–36.65%)	51.73% (44.90–58.49%)	41.92% (36.43–47.63%)	60.13% (54.23–65.76%)	75.54% (69.79–80.50%)
Yes, I drive myself: % (95% CI)	59.12% (53.69–64.35%)	45.36% (39.35–51.51%)	22.99% (18.09–28.75%)	28.67% (23.88–34.00%)	9.66% (6.58–13.95%)	2.04% (0.88–4.65%)
Yes, as a passenger: % (95% CI)	11.70% (8.65–15.64%)	16.61% (12.33–22.02%)	23.82% (18.52–30.07%)	23.06% (18.78–27.97%)	29.31% (24.25–34.93%)	21.58% (16.90–27.12%)
Yes, as driver and passenger: % (95% CI)	9.58% (6.64–13.63%)	7.53% (4.99–11.21%)	1.46% (0.54–3.92%)	6.35% (4.03–9.85%)	0.90% (0.33–2.42%)	0.85% (0.21–3.38%)

### Regression analysis

Table 3 displays results of multinomial logit regression analyses with driving status (no; yes, I drive myself; yes, as a passenger; yes, as driver and passenger) as outcome measure, with no (i.e., not driving a car) as base outcome. Relative risk ratios were reported. Pseudo *R*^2^ was 0.25.

**Table 3:** Correlates of driving status

Independent variables	Yes, I drive myself	Yes, as a passenger	Yes, as driver and passenger
Sex: women (ref.: men)	0.13***	1.10	0.24***
	(0.09–0.18)	(0.83–1.45)	(0.14–0.42)
Age	0.88***	0.98	0.87***
	(0.84–0.91)	(0.95–1.01)	(0.81–0.94)
Marital status: married (Ref.: Married, living separated from spouse; widowed; divorced; single)	1.48*	1.91***	3.31***
	(1.08–2.02)	(1.43–2.55)	(1.89–5.77)
Living situation: living in an institutionalized setting (Ref.: Living in a private household)	0.04**	0.36***	0.34
	(0.01–0.35)	(0.21–0.60)	(0.04–2.76)
Self-rated health (ranging from 1 = very bad to 4 = very good)	1.26*	1.32**	1.75**
	(1.02–1.56)	(1.10–1.57)	(1.20–2.56)
Functional impairment (IADL; ranging from 0 to 2, with higher values corresponding to lower functional impairment)	19.82***	0.99	16.52***
	(12.83–30.62)	(0.81–1.21)	(6.79–40.15)
Number of chronic conditions (ranging from 0 to 19)	1.06 +	1.07*	1.15*
	(0.99–1.14)	(1.01–1.13)	(1.02–1.29)
Observations	1,832	1,832	1,832
Pseudo *R*^2^	.25	.25	.25

Results of multinomial logistic regressions (base outcome: No)

Relative risk ratios are reported, 95% confidence intervals in parentheses; ***p < 0.001, **p < 0.01, * *p* < 0.05, + *p* < 0.10

Regressions showed that being male (RRR: 0.13, 95% CI 0.09–0.18), younger age (RRR: 0.88, 95% CI 0.84–0.91), being married and living together with spouse (RRR: 1.48, 95% CI 1.08–2.02), living in a private household (RRR: 0.04, 95% CI 0.01–0.35), better self-rated health (RRR: 1.26, 95% CI 1.02–1.56), and lower functional impairment (RRR: 19.82, 95% CI 12.83–30.62) were positively associated with ‘Yes, I drive myself’ (compared to not driving a car).

Moreover, being married and living together with spouse (RRR: 1.91, 95% CI 1.43–2.55), living in a private household (RRR: 0.36, 95% CI 0.21–0.60), better self-rated health (RRR: 1.32, 95% CI 1.10–1.57), and a higher number of chronic conditions (RRR: 1.07, 95% CI 1.01–1.13) were positively associated with ‘Yes, as a passenger’ (compared to not driving a car).

Additionally, being male (RRR: 0.24, 95% CI 0.14–0.42), younger age (RRR: 0.87, 95% CI 0.81–0.94), being married and living together with spouse (RRR: 3.31, 95% CI 1.89–5.77), better self-rated health (RRR: 1.75, 95% CI 1.20–2.56), lower functional impairment (RRR: 16.52, 95% CI 6.79–40.15), and a higher number of chronic conditions (IRR: 1.15, 95% CI 1.02–1.29) were positively associated with ‘Yes, as driver and passenger’ (compared to not driving a car).

We trichotomized the outcome (0 = no; 1 = yes, I drive myself or yes, as driver and passenger; 2 = yes, as a passenger) in a sensitivity analysis (Table 4). However, compared to our main regression analysis (presented in Table 3), these findings are quite similar. Please see Table 4 for further details.

**Table 4:** Correlates of driving status

Independent variables	Yes, I drive myself/yes as driver and passenger	Yes, as a passenger
Sex: women (ref.: men)	0.14***	1.09
	(0.10–0.19)	(0.83–1.45)
Age	0.88***	0.98
	(0.84–0.91)	(0.95–1.01)
Marital status: married (Ref.: Married, living separated from spouse; widowed; divorced; single)	1.64**	1.90***
	(1.21–2.23)	(1.43–2.54)
Living situation: living in an institutionalized setting (Ref.: Living in a private household)	0.08**	0.36***
	(0.02–0.36)	(0.21–0.60)
Self-rated health (ranging from 1 = very bad to 4 = very good)	1.31*	1.31**
	(1.07–1.62)	(1.10–1.57)
Functional impairment (IADL; ranging from 0 to 2, with higher values corresponding to lower functional impairment)	19.32***	0.99
	(12.76–29.27)	(0.81–1.21)
Number of chronic conditions (ranging from 0 to 19)	1.07*	1.07*
	(1.00–1.15)	(1.01–1.13)
Observations	1,832	1,832
Pseudo *R*^2^	.28	.28

Results of multinomial logistic regressions (base outcome: No)

Relative risk ratios are reported; 95% confidence intervals in parentheses; ***p < 0.001, **p < 0.01, * *p* < 0.05, + *p* < 0.10

## Discussion

### Main findings

Our goal was to explore the frequency and correlates of driving status among the oldest old. Using data from a large, representative sample, our study showed that about 35% of the individuals aged 80 years and above still drove by themselves [i.e., including (i) individuals who drive by themselves as well as (ii) individuals who drive by themselves and as a passenger]. Beyond that, approximately two out of ten individuals still drove as a passenger. Additionally, regressions particularly showed that driving by oneself was particularly related to sex, age, living situation, and functional impairment (with the expected signs).

### Possible explanations and relation to previous studies

With regard to the frequencies, the proportion of older women still driving a car identified in our study was markedly lower compared to a former study focusing on Australian women aged 85–90 years (worth repeating about 36% in the former study) [9]. We assume that this difference is mainly driven by cultural factors between the countries and perhaps infrastructural factors which may, for example, reflect differences in the necessity of a car for activities of daily life (such as distance to doctors and grocery shops or distance to friends and relatives). Moreover, it may be worth noting that a former German study (mean age of 90.3 years, including GP patients aged 85 years and over) found that 16% still drove a car [8]. This is roughly comparable to our findings in the higher age brackets. More precisely, when we restrict our study to individuals aged 85 years, we found that about 19% of the individuals drove a car by themselves in our study (again, including individuals who drive by themselves as well as individuals who drive by themselves and as a passenger).

Expectedly, we found that being younger and being male were associated with a higher likelihood of driving by oneself (compared to not driving a car). One way to explain such gender-related result is that men more often may view driving a car as a necessity. In contrast, it has been shown that women sometimes avoid stress caused by traffic [17]. Moreover, one can assume that women in this age bracket had a higher likelihood of never driving a car—which would reflect a more classical distribution of roles and which would be in line with former research [18]. Furthermore, women and men differ in terms of risk attitudes of [19] and confidence in their driving skills [20].

In line with former research [21], we found an association between younger age and a higher likelihood of driving by oneself. Beyond the impact of age on the health-related factors which were included in regression analysis, higher age may also reflect an increased awareness of actual driving skills. Thus, oldest old individuals may cease to drive a car. Moreover, oldest old individuals may particularly fear the (perhaps more serious) health consequences of a traffic injury when they drive by themselves and may thus avoid to drive any longer.

Former research has been demonstrated that individuals living in institutionalized settings generally score lower in various health-related factors [22] and also in psychosocial factors [23] such as satisfaction with life. Thus, an association between living arrangement and driving status is very plausible.

Moreover, and in accordance with prior research [8], we found an association between lower functional impairment and a higher likelihood of driving a car by oneself. Such result may be explained by the fact that driving a car reflects a process which is quite complex covering executive functions and reaction [24] as well as visual skills [25] (which are in turn associated with functional impairment [26]).

### Strengths and limitations

This is one of a few studies examining the frequency and correlates of driving status among the oldest old. Data were used from a large, representative sample of individuals residing in North Rhine-Westphalia (both, including community-dwelling and also institutionalized individuals). Established and valid tools were used to quantify the independent variables. This study has a cross-sectional design—which is worth keeping in mind when interpreting the results regarding directionality. Furthermore, the response rate was about 23%. Nevertheless, the NRW80 + is acknowledged as representative for the oldest old residing in North Rhine-Westphalia [15]. Additionally, it should be noted that North Rhine-Westphalia is by far the most densely populated of the “Flächenländer” (area states) in Germany. Consequently, future research among the oldest old individuals living in very rural areas is clearly required. Moreover, upcoming studies could explore further details (e.g., distance driven by own car per year, frequency of driving a car per week).

## Conclusion

A sizable proportion of the individuals aged 80 years and above still drove by themselves. Less than half of the oldest old individuals did not drive. Moreover, our current study identified some correlates of driving status among individuals in latest life. Upcoming research is needed to identify the factors that contribute to driving cessation among the oldest old.
